# Real-world experience of afatinib as a first-line therapy for advanced *EGFR* mutation-positive lung adenocarcinoma

**DOI:** 10.18632/oncotarget.19563

**Published:** 2017-07-26

**Authors:** Sheng-Kai Liang, Min-Shu Hsieh, Meng-Rui Lee, Li-Ta Keng, Jen-Chung Ko, Jin-Yuan Shih

**Affiliations:** ^1^ Department of Internal Medicine, National Taiwan University Hospital Hsinchu Branch, Hsinchu, Taiwan; ^2^ Department of Pathology, National Taiwan University Hospital and College of Medicine, National Taiwan University, Taipei, Taiwan; ^3^ Department of Internal Medicine, National Taiwan University Hospital and College of Medicine, National Taiwan University, Taipei, Taiwan

**Keywords:** afatinib, EGFR mutation-positive, first-line therapy, lung adenocarcinoma, real-world study

## Abstract

We evaluated the real-world efficacy and side effects of afatinib as a first-line therapy for advanced *EGFR* mutation-positive lung adenocarcinoma. The medical records of patients receiving afatinib as a first-line therapy after National Health Insurance reimbursement between May 2014 and January 2016 were reviewed, and information on patient characteristics and treatment courses were collected consecutively. Rebiopsy tissue was collected for *EGFR* mutation and MET amplification analyses. MET amplification was detected by fluorescence *in situ* hybridization and immunohistochemistry. In total, 140 patients were enrolled (median follow-up, 18.0 months). No significant differences in side effects, treatment responses, progression-free survival, or brain metastasis control were observed between patients receiving 40 mg versus < 40 mg of afatinib during the first 6 months. Patients with significant pretreatment weight loss (> 10.0% in 6 months) had a shorter median progression-free survival. Patients with brain metastases had a poorer Eastern Cooperative Oncology Group performance status and were associated with a shorter median progression-free survival. Nine patients (32.1%) had a p.T790M mutation and only 1 patient gained MET amplifications after disease progression. Afatinib is effective as a first-line therapy for advanced *EGFR* mutation-positive lung adenocarcinoma. Afatinib dosage does not affect clinical efficacy and drug-related side effects.

## INTRODUCTION

Lung adenocarcinoma, the major histological subtype of non-small cell lung cancer, has been further subclassified into several molecular subsets with susceptibility to specific ‘‘targeted’’ drugs as advances have been made in translational research. Mutations in the *EGFR* kinase domain could activate downstream signaling pathways and cause cancer cells to proliferate, metastasize, and invade other tissues, or become resistant to apoptosis [1–3]. Two major *EGFR* mutations, exon 19 deletions and exon 21 p.L858R point mutation, account for approximately 90.0% of *EGFR* mutations [4]. These are referred to as “classical” *EGFR* mutations and are well documented for effectively responding to epidermal growth factor receptor-tyrosine kinase inhibitor (EGFR-TKI) treatment. Notably, the use of first-generation EGFR-TKIs (e.g., gefitinib or erlotinib) as a first-line therapy for patients with advanced *EGFR* mutation-positive lung adenocarcinoma has been associated with a higher objective response rate (ORR) and longer progression-free survival (PFS) than platinum-based doublet chemotherapy [5, 6].

Afatinib, an irreversible second-generation EGFR-TKI and member of the ErbB family, has activity against not only “classical” *EGFR* mutations, but also rare *EGFR* mutations, including the exon 18 p.G719X and exon 21 p.L861Q point mutations [7–9]. Afatinib has also proven to be effective as a first-line therapy in patients with advanced *EGFR* mutation-positive lung adenocarcinoma in the LUX-Lung 3 [10] and LUX-Lung 6 [11] trials. In these phase III trials [10, 11] afatinib was associated with a significantly prolonged PFS compared to first-line chemotherapy with cisplatin and pemetrexed or cisplatin and gemcitabine, respectively. In a subsequent phase IIB trial (LUX-Lung 7 [12]) afatinib conferred a significant benefit in prolonging PFS and time to treatment failure, but not overall survival (OS), compared to first-line treatment with gefitinib in patients with advanced *EGFR* mutation-positive lung adenocarcinoma.

These well designed randomized controlled trials emphasize the efficacy and safety of the study drugs in an extremely controlled environment and patient population. However, drug effectiveness may be confounded by several factors (e.g., patient group selection, comorbidities, adherence, and other organizational factors). The real-world experiences reflect these variations in influential factors that may be excluded from randomized controlled trials [13]. Meanwhile, the tolerability of afatinib-related side effects, the efficacy following various clinical adjustments, and the mechanism(s) of acquired resistance have yet to be determined in the post-approval period.

In this study, we analyzed a real-world cohort of afatinib-treated patients from a tertiary medical center in Taiwan and consecutively investigated the efficacy and side effects of afatinib by reviewing patients’ medical records. Furthermore, we evaluated the mechanism(s) of acquired resistance of afatinib from rebiopsy tissue after disease progression.

## RESULTS

### Patient characteristics and clinical response to afatinib

We retrospectively retrieved the study cohort from an approved list of afatinib applications to the Taiwan National Health Insurance scheme at the National Taiwan University Hospital (Taipei, Taiwan) between May 2014 and January 2016. In total, 140 patients with advanced *EGFR* mutation-positive lung adenocarcinoma who had received afatinib as a first-line treatment were enrolled in this study. The median age of the patients was 61 (range, 28–87) years. Eighty-seven patients (62.1%) were women and 98 patients (70.0%) had never smoked. The clinical characteristics of patients were listed in Table 1. Patients were stratified into three groups according to their *EGFR* mutation status: Group 1, “classical” mutation; Group 2, complex mutation with classical mutation; and Group 3, rare mutation with or without complex mutation (Table 1).

**Table 1:** Clinical characteristics and comparison of patient groups according to afatinib treatment in the first 6 months

Characteristic	Afatinib-treated patients in the first 6 months			*P*-value
	All (*n* = 140)	40 mg (*n* = 81)	< 40 mg (*n* = 59)	
Age (years), median (range)	61 (28–87)	61 (28–82)	63 (33–87)	
Sex, *n* (%)				0.060
M	53 (37.9)	36 (44.4)	17 (28.8)	
F	87 (62.1)	45 (55.6)	42 (71.2)	
Smoking status, *n* (%)				0.450
Never smoked	98 (70.0)	54 (66.7)	44 (74.6)	
Ex-smoker^a^	19 (13.6)	11 (13.6)	8 (13.5)	
Current smoker	23 (16.4)	16 (19.7)	7 (11.9)	
BMI, mean (SD)^b^	23.4 (3.2)	23.8 (3.2)	22.8 (3.1)	0.058
BSA, mean (SD)^c^	1.62(0.14)	1.65(0.17)	1.58(0.14)	0.067
Weight loss at diagnosis, *n*(%)				0.661
≤10.0%	109 (77.9)	62 (76.5)	47 (79.7)	
> 10.0%^d^	31 (22.1)	19 (23.5)	12 (20.3)	
Baseline ECOG PS, *n*(%)				0.527
0–1	129 (92.1)	76 (93.8)	53 (89.8)	
2–4	11 (7.9)	5 (6.2)	6 (10.2)	
cStage at screening, *n*(%)				> 0.999
Stage IIIB	4 (2.9)	2 (2.5)	2 (3.4)	
Stage IV	136 (97.1)	79 (97.5)	57 (96.6)	
Metastatic site at screening, *n*(%)				
Lung	73 (52.1)	39 (48.1)	34 (57.6)	0.268
Bone	48 (34.3)	29 (35.8)	19 (32.2)	0.658
Brain	42 (30.0)	24 (29.6)	18 (30.5)	0.911
Liver	12 (8.6)	7 (8.6)	5 (8.5)	0.972
Adrenal glands	9 (6.4)	4 (4.9)	5 (8.5)	0.493
Other	15 (10.7)	7 (8.6)	8 (13.6)	0.353
*EGFR* mutation status,*n* (%)				0.006^*^
Group 1 (classical mutation[s])	108 (77.1)	70 (86.4)	38 (64.4)	
19DEL	81 (57.9)	50 (61.7)	31 (52.5)	
p.L858R	24 (17.1)	18 (22.3)	6 (10.2)	
p.L858R and 19DEL	3 (2.1)	2 (2.5)	1 (1.7)	
Group 2 (complex mutation with classical mutation)	6 (4.3)	3 (3.7)	3 (5.1)	
p.L858R and p.T790M	4 (2.9)	2 (2.5)	2 (3.4)	
Other	2 (1.4)	1 (1.2)	1 (1.7)	
Group 3 (Rare mutation with or without complex mutation)	26 (18.6)	8 (9.9)	18 (30.5)	
p.L861Q	10 (7.1)	1 (1.2)	9 (15.2)	
p.G719A	6 (4.3)	3 (3.7)	3 (5.1)	
20-INS	4 (2.9)	0 (0.0)	4 (6.8)	
p.G719A and p.T790M/Other	6 (4.3)	4 (4.9)	2 (3.4)	

^*^ *P* < 0.05

^a^Ceased smoking > 1 year before diagnosis

^b^BMI = body weight (kg)/body height (m)^2^

^c^BSA= [body Height (cm) x body weight (kg)/ 3600 ]½

^d^Significant weight loss of >10.0% within 6 months of diagnosis

BMI, body mass index; BSA, body surface area; cStage, clinical stage; DEL, deletion; ECOG, Eastern Cooperative Oncology Group; F, female; INS, insertion; M, male; PS, performance status; SD, standard deviation

After afatinib administration, 99 patients (70.7%) experienced Grade ≥ 2 skin lesions and were referred to a dermatologist for further care and 32 patients (22.9%) experienced Grade ≥ 2 diarrhea and were treated with intermittent antidiarrheal agents (Table 2). Notably, 7 patients (5.0%) discontinued afatinib treatment because of severe side effects. In these patients, despite its clinical effectiveness, afatinib was switched to gefitinib or erlotinib (Supplementary Table 1).

**Table 2:** Comparison of effects and side effects according to afatinib treatment in the first 6 months

Variable	Afatinib-treated patients in the first 6 months			*P*-value
	All (*n* = 140)	40 mg (*n* = 81)	< 40 mg (*n* = 59)	
Side effects of afatinib treatment, *n* (%)				
Skin lesion of ≥ Grade 2	99 (70.7)	57 (70.4)	42 (71.2)	0.917
Diarrhea of ≥ Grade 2	32 (22.9)	18 (22.2)	14 (23.7)	0.834
Tumor response to afatinib treatment, *n* (%)				0.087
PR	94 (67.2)	60 (74.1)	34 (57.6)	
SD	37 (26.4)	18 (22.2)	19 (32.2)	
PD	9 (6.4)	3 (3.7)	6 (10.2)	
Weight loss of > 5.0% during afatinib treatment, *n* (%)	66 (47.1)	39 (48.2)	27 (45.8)	0.545
PD event, *n* (%)	77 (55.0)	43 (53.1)	34 (57.6)	0.594
PD due to brain event, *n* (%)	26 (18.6)	13 (16.1)	13 (22.0)	0.540

PD, progressive disease; PR, partial response; SD, stable disease

Treatment responses to afatinib were determined through imaging studies and by reviewing patients’ medical records (Table 2). Ninety-four patients (67.2%) exhibited a partial response, 37 patients (26.4%) had stable disease, and 9 patients (6.4%) had progressive disease. Sixty-six patients (47.1%) experienced weight loss of > 5.0% during the whole afatinib treatment course. The median PFS of the total patient population was 11.8 (95.0% confidence interval [CI]: 10.9–13.0) months. Patients with a partial response after afatinib treatment had a longer PFS compared to those with stable or progressive disease (12.8 [95.0% CI: 9.5–16.1] *vs.* 8.0 [95.0% CI: 5.9–10.1] months, respectively; *P* = 0.001).

### Dose adjustments of afatinib in a real-world cohort

The conditions of the dose adjustments in our real-world cohort were recorded (Supplementary Table 2). Ninety-eight patients (70.0%) received afatinib at an initial dose of 40 mg. Of these, 29 patients (29.6%) underwent dose reduction. The remaining 42 patients (30.0%) received afatinib at an initial dose of 30 mg. Of these, 9 patients (21.4%) underwent dose reduction. In 25 (65.8%) of the 38 patients, the dose of afatinib was adjusted during the first 6 months of treatment. In 10 patients, further dose adjustments were made, including dose escalation in 6 patients.

### Afatinib doses of < 40 mg in the first 6 months do not influence clinical efficacy

**Figure 1:**
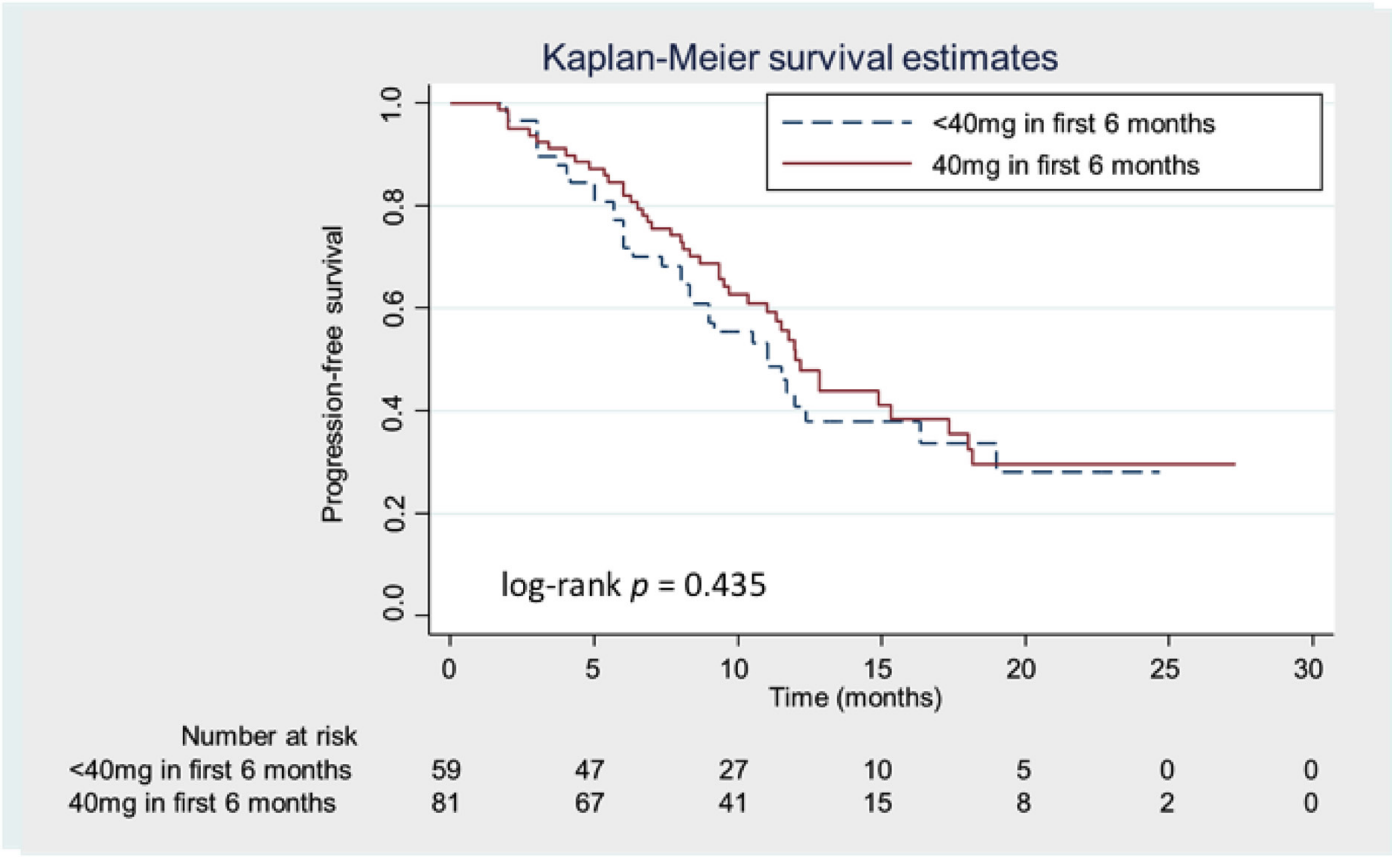
Kaplan-Meier progression-free survival (PFS) curves according to the treatment dose of afatinib Patients receiving 40 mg and < 40 mg of afatinib during the first 6 months are represented by the solid (red) and dashed (blue) lines, respectively.

Most patients received dose adjustments during the first 6 months in LUX-Lung 3/6 [17] and our current study, and then patients were subdivided into two groups according to the dose of afatinib during the first 6 months of treatment (40 mg *versus* < 40 mg). No significant differences in various clinical characteristics, including age, smoking status, ECOG PS, and the sites of metastases, were observed between the two groups. There were trends towards male gender, a BMI of ≥ 20.0, and higher BSA being associated with a relatively high tolerability to 40 mg of afatinib during the first 6 months (Table 1). However, a greater proportion of patients had “classical” mutations in the 40 mg group than the < 40 mg group (*P* = 0.006). No significant differences in afatinib-related side effects or treatment responses were observed between the 40 mg and < 40 mg groups (Table 2). There was also no significant difference in the median PFS between the 40 mg and < 40 mg groups (12.0 *vs.* 11.0 months, respectively; *P* > 0.05 [Figure 1]) (hazard ratio [HR]: 0.84 [95.0% CI: 0.53–1.31]).

### Patients with brain metastases were associated with unfavorable outcomes

**Figure 2:**
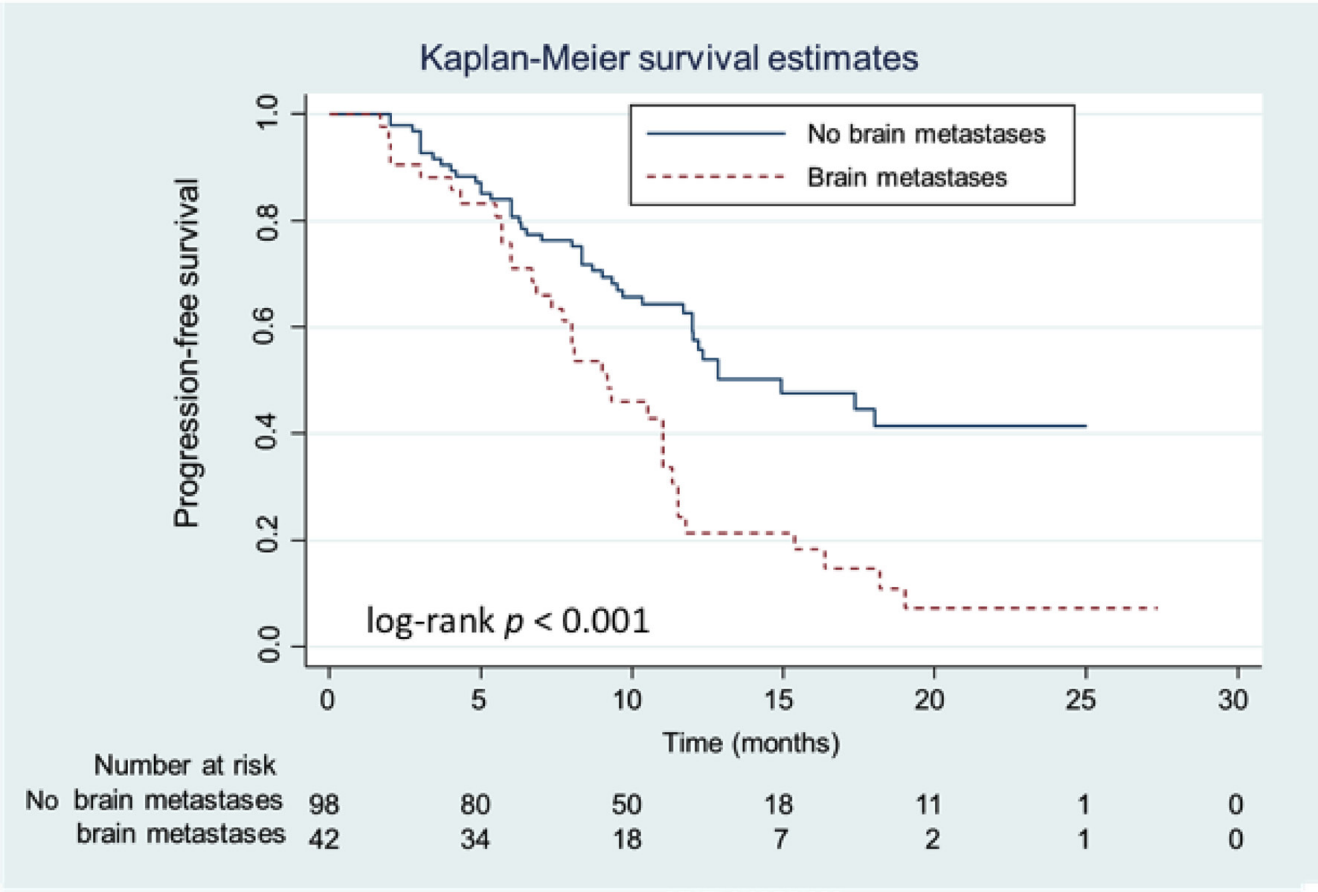
Kaplan-Meier progression-free survival (PFS) curves of patients with and without brain metastases Patients with and without brain metastases are represented by the dashed (red) and solid (blue) lines, respectively.

**Figure 3:**
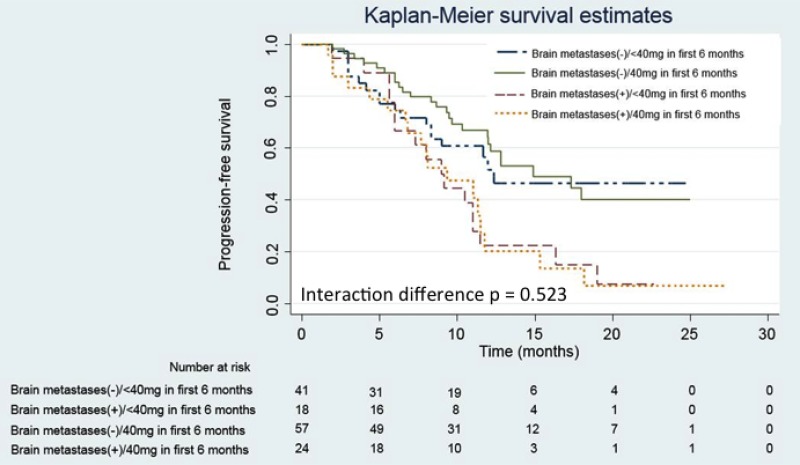
Kaplan-Meier progression-free survival (PFS) curves of patients with and without brain metastases according to the treatment dose of afatinib Patients with brain metastases receiving 40 mg and < 40 mg of afatinib during the first 6 months are represented by the dashed (yellow) and dashed (red) lines, respectively. Patients without brain metastases receiving 40 mg and < 40 mg of afatinib during the first 6 months are represented by the solid (green) and dashed (blue) lines, respectively.

Advanced lung adenocarcinoma patients frequently present with brain metastases at the time of diagnosis. In our real-world cohort, 42 patients (30.0%) presented with brain metastases at the time of initial diagnosis. Patients with brain metastases were initially associated with a poorer ECOG PS compared to patients without brain metastases (*P* = 0.003; Table 3). However, *EGFR* mutation patterns were not clinically associated with brain metastases. At the data cutoff date, patients with brain metastases suffered from more progressive events (*P* < 0.001), especially progression of their brain tumors (*P* = 0.023). Patients with initial brain metastases were associated with a shorter median PFS than those without brain metastases (9.2 *vs.* 14.9 months, respectively; *P* < 0.001 [Figure 2]) (HR: 2.29 [95.0% CI: 1.46–3.60]). However, no interaction in the median PFS was observed between the brain metastases and afatinib dosage groups (Figure 3), suggesting that the median PFS of patients with brain metastases is not influenced by an afatinib dose of < 40 mg.

**Table 3:** Clinical characteristics of patients with or without brain metastases (BMs)

Characteristic	Patients with BMs (*n* = 42)	Patients without BMs (*n* = 98)	*P*-value
Age (years), *n* (%)			0.970
< 65	26 (61.9)	61 (62.2)	
≥ 65	16 (38.1)	37 (37.8)	
Sex, *n* (%)			0.732
M	15 (35.7)	38 (38.8)	
F	27 (64.3)	60 (61.2)	
BMI, *n* (%)^a^			0.405
< 20	4 (9.5)	14 (14.3)	
≥ 20	38 (90.5)	84 (85.7)	
Weight loss at diagnosis, *n* (%)			0.756
≤ 10.0%	32 (76.2)	77 (78.6)	
> 10.0%^b^	10 (23.8)	21 (21.4)	
ECOG PS, *n* (%)			0.003^*^
0–1	34 (81.0)	95 (96.9)	
2–4	8 (19.0)	3 (3.1)	
*EGFR* mutation status, *n* (%)			0.921
Group 1^c^	33 (78.5)	75 (76.5)	
Group 2^d^	2 (4.8)	4 (4.1)	
Group 3^e^	7 (16.7)	19 (19.4)	
Weight loss of >5.0% during afatinib treatment, *n* (%)	25 (59.5)	41 (41.8)	0.066
PD event, *n* (%)	34 (81.0)	43 (43.9)	< 0.001^*^
PD due to brain event, *n* (%)	16 (38.1)	10 (10.2)	0.023^*^

^*^ *P* < 0.05

^a^BMI = body weight (kg)/body height (m)^2^

^b^Significant weight loss of > 10.0% within 6 months of diagnosis

^c^Classical mutation(s)

^d^Complex mutation with classical mutation

^e^Rare mutation with or without complex mutation

BMI, body mass index; ECOG, Eastern Cooperative Oncology Group; F, female; M, male; PD, progressive disease; PS, performance status

### Treatment responses in *EGFR* mutation subgroups after afatinib treatment

**Figure 4:**
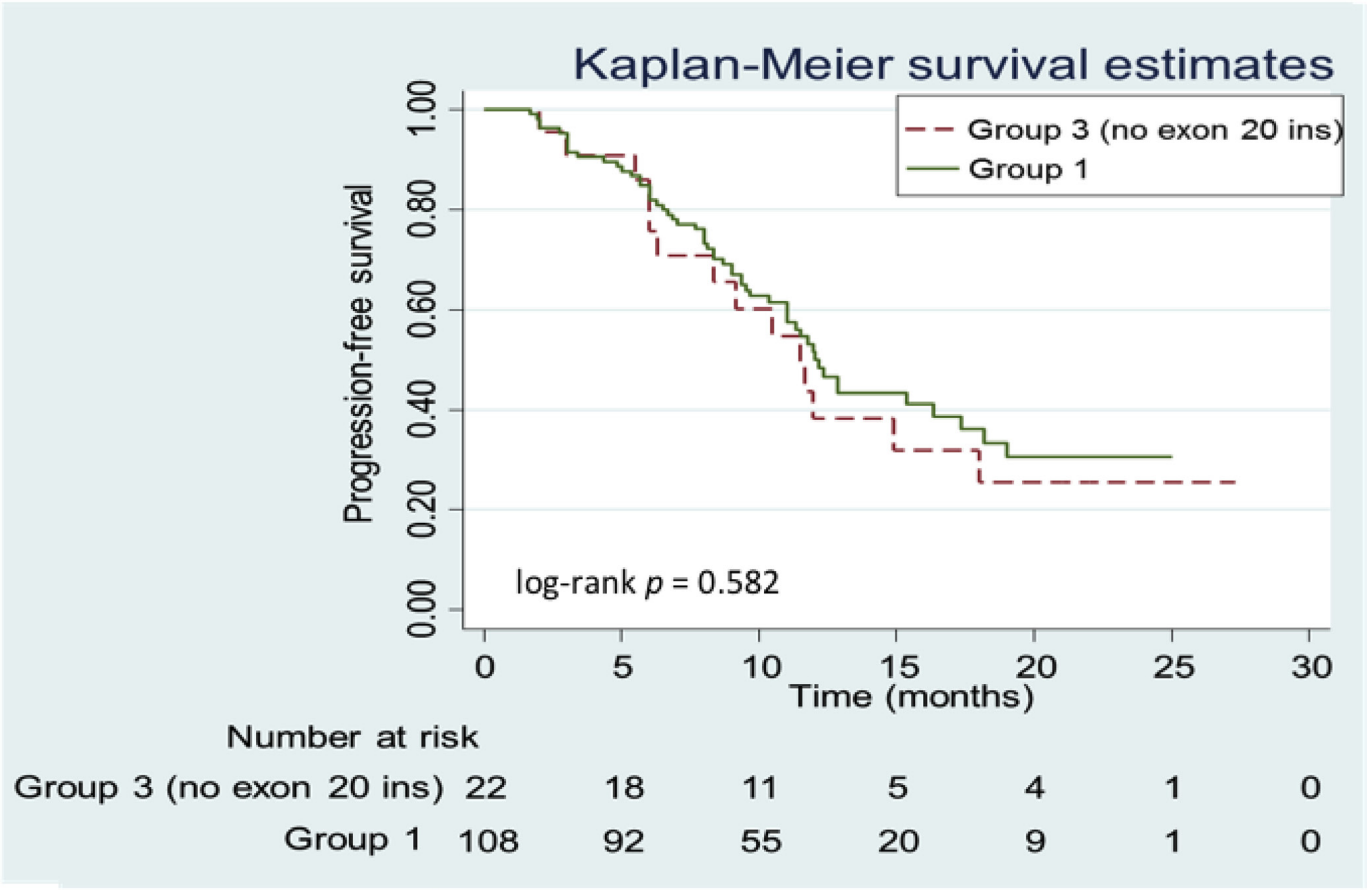
Kaplan-Meier progression-free survival (PFS) curves of patients in Group 1 *vs*. Group 3 (no exon 20 insertion [20-INS]) Patients in Group 1 are represented by the solid (green) line and patients in Group 3 are represented by the dashed (red) line.

**Figure 5:**
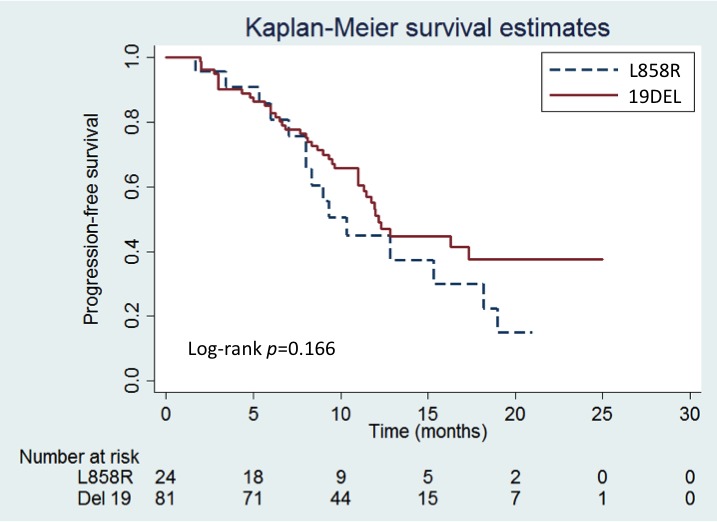
Kaplan-Meier progression-free survival (PFS) curves of patients with an exon 19 deletion (19DEL) *vs*. p.L858R mutation Patients with the 19DEL are represented by the solid (red) line and patients with the p.L858R mutation are represented by the dashed (blue) line.

Among the *EGFR* mutation subgroups, Group 1 and Group 2 patients were associated with favorable ORRs of 70.4% and 66.7%, respectively (Supplementary Table 3), while Group 3 patients were associated with a lower ORR of 53.9% (*P* > 0.05). For the 5 patients with a primary p.T790M mutation (p.T790M and p.L858R [*n* = 4] and p.T790M and p.G719A [*n* = 1]), the ORR was 60.0%. For the 4 patients with an exon 20 insertion, the ORR was 25.0% (partial response [*n* = 1], stable disease [*n* = 2], and progressive disease [*n* = 1]). The median PFS of the Group 1 patients (“classical” *EGFR* mutations) was 12.2 months, while the median PFS of the Group 2 and Group 3 patients was 9.2 months. The tumors of patients with exon 20 insertions were well known to be resistant to EGFR-TKI treatment. After excluding those patients with exon 20 insertions from subsequent analyses, no significant difference in the median PFS was observed between Group 1 and Group 3 patients (12.2 *vs.* 11.5 months, respectively; *P* > 0.05 [Figure 4]) (HR: 0.85 [95.0% CI: 0.47–1.53]). There was also no significant difference in the median PFS between the 81 patients with exon 19 deletions and the 24 patients with p.L858R point mutations (12.2 *vs.* 10.3 months, respectively; *P* > 0.05 [Figure 5]) (HR: 0.66 [95.0% CI: 0.36–1.20]).

### Univariate and multivariate analyses of clinical factors associated with progression-free survival

In order to realize the effects of clinical factors on the PFS of afatinib-treated patients in a real-world cohort, we performed univariate and multivariate analyses. Both univariate and multivariate analyses revealed that patients presenting with significant weight loss (>10.0%) and brain metastases were associated with a significantly shorter PFS (Table 4). Therefore, treatment responses at initial evaluation could provide the reference for a longer treatment benefit.

**Table 4:** Univariate and multivariate analyses of clinical factors for progression-free survival in a real-world cohort

Clinical factor	Patients (*n*)	Univariate analysis		Multivariate analysis	
		HR (95.0% CI)	*P*-value	HR (95.0% CI)	*P*-value
Age (years)					
< 65	87	1		–	
≥ 65	53	1.39 (0.89–2.19)	0.153	–	–
Sex					
M	53	1.07 (0.68–1.69)	0.776	–	–
F	87	1		–	
Smoking status					
Never smoked	98	1		–	
Ex-smoker	19	1		–	
Current smoker	23	1.18 (0.86–1.60)	0.304	–	–
BMI					
< 20	18	1.33 (0.72–2.46)	0.371	–	–
≥ 20	119	1		–	
Weight loss at diagnosis					
≤ 10.0%	109	1		1	
> 10.0%	31	1.73 (1.04–2.88)	0.035^*^	1.75 (1.05–2.93)	0.033^*^
ECOG PS					
0–1	129	1		1	
2–4	11	2.15 (1.02–4.52)	0.043^*^	1.48 (0.69–3.16)	0.310
BMs					
Present	42	2.29 (1.46–3.60)	< 0.001^*^	2.09 (1.32–3.32)	0.002^*^
Absent	98	1		1	
*EGFR* mutation status					
Group 1^a^	108	0.68 (0.41–1.13)	0.130	0.67 (0.41–1.12)	0.125
Group 2–3^b,c^	32	1		1	
Tumor response to afatinib treatment					
PR	94	0.49 (0.31–0.77)	0.002^*^	0.49 (0.31–0.79)	0.003^*^
SD/PD	46	1		1	
Afatinib dose during the first 6 months of treatment (mg)					
40	81	0.84 (0.53–1.31)	0.435	–	–
< 40	59	1		–	

^*^ *P* < 0.05

^a^Classical mutation(s)

^b^Complex mutation with classical mutation

^c^Rare mutation with or without complex mutation

BM, brain metastasis; BMI, body mass index; CI, confidence interval; ECOG, Eastern Cooperative Oncology Group; F, female; HR, hazard ratio; M, male; PD, progressive disease; PR, partial response; PS, performance status; SD, stable disease

### Mechanism(s) of acquired resistance to afatinib

Twenty-eight patients underwent rebiopsy (lung tumor biopsy [*n* = 14], lymph node biopsy [*n* = 3], liver tumor biopsy [*n* = 2], and brain tumor excision [*n* = 1]), malignant pleural effusion drainage (*n* = 6), or liquid biopsy (*n* = 2) after acquiring resistance to afatinib. Seventeen of the 20 rebiopsy specimens were adequate for MET immunohistochemical (IHC) staining (Supplementary Table 4). Confirmation of MET amplification by fluorescence *in situ* hybridization (FISH) was performed only in samples with MET positivity on immunohistochemistry (2+ or 3+ staining intensity in >50.0% of the tumor cells according to the Metmab criteria[14]). Of the 28 patients who underwent rebiopsy, 9 patients (32.1%) had a p.T790M mutation (6 from tumor tissues, 2 from pleural effusions, and one from liquid biopsy). Patients who gained a p.T790M mutation after acquiring resistance to afatinib exhibited a trend towards a longer median PFS compared to those who had not (9.3 *vs.* 7.3 months, respectively; *P* > 0.05) (HR: 0.57 [95.0% CI: 0.24–1.33]). Seven of 20 samples had MET positivity on immunohistochemistry. Furthermore, FISH only confirmed one positive MET amplifications. We also checked the MET IHC staining and amplification from the paired specimens before afatinib treatment. Totally 16 paired specimens were analyzed. Five (31.3%) of 16 pre-afatinib tissues had MET positivity on IHC, but none of them had MET amplification by FISH. Comparing the paired tissue, 4 tumors with grade 0~1+ of MET IHC staining in pre-afatinib biopsy became grade 2+~3+ in rebiopsy specimens, and 7 grade 0~1+ tumors did not change their MET expression. Conversely, 3 samples with grade 2~3+ staining before afatinib becoming grade 0~1+ after treatment. Notably, 2 tumors with MET grade 2+~3+ consistently gained grade 3+ stain in rebiopsy specimens, and one of them gained MET amplification after afatinib resistance.

## DISCUSSION

In this real-world cohort study, the enrolled patients had a relatively good ECOG PS, with 129 (92.1%) of 140 patients having an ECOG PS of 0–1. This was comparable to the LUX-Lung 3 [10] and LUX-Lung 6 [11] trials in which only patients with a favorable ECOG PS of 0–1 were enrolled. The median age of our patients was 61 years, which was also comparable to the LUX-Lung 3 [10] and LUX-Lung 6 [11] trials, with a median age of 62 and 58 years, respectively. Ninety-eight (70.0%) of the 140 enrolled patients had never smoked, which was comparable to the 67.4% and 74.8% in the LUX-Lung 3 [10] and LUX-Lung 6 [11] trials, respectively. The median PFS of our patients (11.8 months) was also comparable to that of the LUX-Lung 3 [10] and LUX-Lung 6 [11] trials (11.0 and 11.1 months, respectively).

Dose prescriptions and adjustments of afatinib are of considerable clinical concern owing to the tolerability of adverse events, with most drug adjustments having been made during the first 6 months in clinical trials [15, 16]. In our study, afatinib was prescribed at an initial dose of 40 mg in 70.0% of the patient population. Of these, 29.6% of patients underwent dose reduction (the majority within the first 6 months). Although a minority of the patient population had received afatinib at an initial lower dose of 30.0 mg, 21.4% of patients still underwent dose reduction. In 7 patients, afatinib was switched to another EGFR-TKI (gefitinib or erlotinib), due to the presence of severe adverse events. None of the patients experiencing diarrhea were switched to a different EGFR-TKI, suggesting that diarrhea could be treated by dose reduction of afatinib and prescription of antidiarrheal agents. In a comparison of the 40 mg and < 40 mg groups, male gender, a BMI of ≥ 20.0, and higher BSA were more prevalent in the 40 mg group. Conversely, no significant difference in age was observed between the two groups.

The LUX-Lung 3 and LUX-Lung 6 post hoc analyses focusing tolerability-guided dose adjustments [17] could reduce afatinib-related adverse events without affecting therapeutic efficacy. In this real-world cohort study, patients administered with 40 mg of afatinib had a higher ORR than patients administered with < 40 mg of afatinib (74.0% *vs.* 58.0%, respectively), although this was not significant (*P* > 0.05). The median PFS was also not significant between the two groups (HR: 0.84 [95.0% CI: 0.53–1.31]; *P* > 0.05).

Subgroup analyses of the LUX-Lung 3 and LUX-Lung 6 trials have demonstrated that lung adenocarcinoma patients harboring exon 19 deletions have a favorable PFS compared to patients harboring p.L858R point mutations. In a molecular epidemiology study focusing on *EGFR* mutations across 7 Asian regions [4], including Taiwan, the proportion of patients with exon 19 deletions and p.L858R point mutations were found to be comparable (22.1% *vs.* 20.9% of 1,450 patients, respectively). In our real-world cohort study, 81 patients (57.9%) with exon 19 deletions and 24 patients (17.1%) with p.L858R point mutations were enrolled, which differed from the proportions of patients in the Asian epidemiological report. One possible explanation could be that physicians have prescribed afatinib as a first-line treatment based on the significantly favorable outcome of OS in the exon 19 deletion subgroup [18, 19]. In our study, we were unable to represent and reproduce the significantly superior OS outcome in exon 19 deletion patients compared to p.L858R point mutation patients owing to the disproportionate patients enrolled and limited follow-up (maximum duration, 28.0 months). Therefore, further clinical studies may be required to determine whether exon 19 deletion patients have a superior PFS outcome after afatinib treatment compared to those harboring the p.L858R point mutation.

Patients harboring mutations of *de novo* p.T790M mutations and exon 20 insertions, have demonstrated a reduced benefit in clinical outcomes compared to other mutations (e.g., p.G719X, p.L861Q, and p.S768I) [20]. In our real-world cohort study, ORRs of 25.0% and 60.0% were observed for patients with exon 20 insertions or complex mutations and *de novo* p.T790M mutations, respectively. These 5 *de novo* p.T790M were detected by the method of matrix-assisted laser desorption ionization–time of flight mass spectrometry (MALDI-TOF MS) and had concomitant p.L858R or p.G719A mutation. The MALDI-TOF MS method was ultra-sensitive to detect minor pre-treated p.T790M than direct sequencing [21]. Despite the initial response to afatinib, 3 of those 5 patients experienced disease progression within 6 months.

EGFR-TKI concentration and penetration of the blood-brain-barrier have remained a concern in treating lung adenocarcinoma patients with brain metastases. High-dose first-generation EGFR-TKIs may be administered as a single agent for brain metastases to increase the cerebrospinal fluid drug concentration [22, 23]. However, the efficacy and survival benefits are unclear due to a lack of conclusive evidence. Nonetheless, not all patients can tolerate the side effects. The concomitant use of erlotinib and whole brain radiotherapy followed by erlotinib maintenance therapy could be advantageous in terms of PFS or OS outcomes in *EGFR* mutation-positive patients [24]. The Afatinib Compassionate Use Consortium [25] has stated that a greater cerebral response to afatinib treatment was observed in 11 (35.5%) of 31 *EGFR* mutation-positive patients compared to those in the chemotherapy group. In our real-world cohort study, patients with brain metastases had a significantly poorer median PFS compared to those without brain metastases.

The mechanism(s) of acquired resistance after receiving afatinib as a first-line treatment for advanced *EGFR* mutation-positive lung adenocarcinoma is not clearly understood. The patients enrolled in this study were all systemic treatment-naïve patients. The most common mechanism of acquired resistance to first-generation EGFR-TKIs is the p.T790M mutation that accounts for 50.0–63.0% of cases [26, 27]. The p.T790M mutation also represents the primary mechanism of acquired resistance to afatinib that accounts for 36.4% (4 of 11 patients having rebiopsy samples) [28] and 50.0% (7 of 14 patients receiving afatinib as first-line treatment) [29]. Our findings confirmed that the p.T790M mutation was the main mechanism of acquired resistance, followed by MET amplifications. Patients with p.T790M mutation-positive acquired resistance had a significantly longer post-progression survival and more indolent progression of the lung adenocarcinoma than p.T790M mutation-negative patients [30, 31]. The limited availability of rebiopsy tissue and shorter follow-up intervals (maximum duration, 28.0 months) has led to a reduction in the incidence of the p.T790M mutation compared to previous studies [28, 29].

This study had some limitations. First, this was a retrospectively observational study. There was a bias of selection of usage of EGFR TKIs. Eight patients with PS 4 received afatinib for less than 30 days were excluded for analysis. Second, we did not perform next-generation sequencing to have a complete analysis of possible acquired resistant mechanism because of the limited tissue and facility.

In conclusion, real-world first-line afatinib data reproduces the findings of several clinical trials. Dose reduction does not reduce the efficacy of afatinib. Patients with brain metastases at the time of initial diagnosis had a poorer ECOG PS, a shorter PFS, and suffered more disease progression in the brain. Advanced *EGFR* mutation-positive non-small cell lung cancer patients presenting with significant weight loss (>10.0%) and brain metastases had a shorter PFS. The treatment response could provide the reference for a longer treatment benefit. The p.T790M mutation is the most common mechanism of acquired resistance to afatinib.

## MATERIALS AND METHODS

### Patients and data collection

Afatinib has been reimbursed by the Taiwan National Health Insurance scheme as a first-line therapy for the treatment of advanced *EGFR* mutation-positive lung adenocarcinoma patients since May 2014. We subsequently retrieved the list of approved afatinib applications to the Taiwan National Health Insurance scheme from the National Taiwan University Hospital (Taipei, Taiwan) between May 2014 and January 2016. Patients were excluded because (1) their *EGFR* mutation status was unknown, (2) they were administered afatinib for < 30 days, (3) they were treated with combination immunotherapy, or (4) they had received palliative chemotherapy prior to afatinib treatment. This study was approved by the Research Ethics Committee of the National Taiwan University Hospital (Taipei, Taiwan). All participants have provided written informed consent and research was conducted in accordance with the 1964 Declaration of Helsinki and its later amendments.

Total 152 patients were screened initially, and 12 patients were excluded from our study, including 4 patients received other EGFR TKI treatment previously or immune checkpoint therapy concomitantly, and 8 patients received afatinib less than 30 days because of PS 4 (Supplementary Figure 1). The 140 enrolled patients’ clinical characteristics and medical records, including demographic information and treatment courses, were collected consecutively until August 2016. Ex-smokers were defined as patients who had ceased smoking for >1 year at the time of a lung cancer diagnosis. Body height and weight were measured and presented as the BMI (body weight [kg]/body height [m]^2^) and BSA ([body Height (cm) x body weight (kg)/ 3600 ]^½^). According to ‘malnutrition universal screening tool’ (‘MUST’) for nutrition evaluation of adult cancer patients, BMI less than 20 when cancer diagnosed was recognized as mild malnutrition status [32]. Poor nutrition status may easily cause these patients to interrupt treatment regimens and have uncontrolled diseases [33]. Therefore, we set the threshold of BMI of 20.0 for pre-treatment nutrition evaluation and possibility of treatment interruptions. Significant weight loss was defined as a >10.0% loss within 6 months prior to the diagnosis of lung cancer. The PS was determined according to the ECOG scale [34]. All lung adenocarcinoma patients had advanced-stage disease (Stage IIIB or Stage IV according to the American Joint Committee on Cancer [seventh edition] [35]) and received single-agent treatment with afatinib. Adverse events were categorized and graded according to the United States National Cancer Institute's Common Terminology Criteria for Adverse Events (version 3.0) [36].

The dosage and duration of afatinib treatment were also recorded. Although BIBW2992 (afatinib) phase I trial proved the safety and antitumor activity of 40 or 50 mg as daily dosage [37], afatinib was approved starting dose 40 mg/day then applying to several clinical trials [10, 11]. Patients who remained on 40 mg of afatinib per day (none of the patients in our hospital exceeded 40 mg) during the first 6 months of treatment were compared with those whose dose was reduced to < 40 mg per day [17].

Tumor responses were determined from patients’ medical records by the primary care physician and independent image reviewing by investigators according to the Response Evaluation Criteria in Solid Tumors (version 1.1) [38]. PFS was defined, in days, as the period from commencing afatinib treatment to the point of radiologically documented disease progression or death from any cause.

Meanwhile, the results of the molecular and pathological analyses were checked against the patients’ medical records.

The cutoff date for acquiring consecutive data and follow-up clinical information after afatinib treatment in this patient population was August 31, 2016. The median follow-up duration was 18.0 (range, 8.0–28.0) months.

### Information on *EGFR* mutation status

We retrospectively collected the records of the *EGFR* mutation status of patients from formal pathology reports and referral data from other hospitals. Cancer specimens from our hospital included primary lung adenocarcinomas, tissues from metastatic sites, and malignant effusion cell blocks.

### Detection of MET expression by immunohistochemical staining

Tissue sections (4.0 μm thick) were dewaxed and rehydrated. Antigen retrieval was performed in an autoclave using Epitope Retrieval Solutions (Leica Biosystems, Newcastle, UK) at pH 9.0 for 10 minutes at 121.0°C. The slides were allowed to react with a c-MET specific antibody (clone SP44, 1:50 dilution; Ventana Medical Systems, Inc., Tucson, AZ, USA) at room temperature for 1 hour. The slides were incubated using an immunohistochemical stain detection kit (UltraVision^TM^ Quanto Detection System; Thermo Fisher Scientific, Inc., Waltham, MA, USA) and counterstained with hematoxylin. For the negative controls, the primary antibody was replaced with 5.0% fetal bovine serum. Staining intensities were categorized as 0 (no staining), 1+ (weak cytoplasmic staining), 2+ (moderate cytoplasmic staining), or 3+ (strong cytoplasmic staining). MET positivity by immunohistochemistry was defined as a 2+ or 3+ staining intensity in ≥ 50.0% of the tumor cells according to the Metmab criteria used in the NCT01456325 study [14], a Metmab phase III trial of advanced non-small cell lung cancer patients.

### Detection of MET amplification by fluorescence *in situ* hybridization

The commercially available ZytoLight SPEC MET/CEN 7 Dual Color Probe (ZytoVision GmbH, Bremerhaven, Germany) was used to detect MET amplifications. Briefly, paraffin-embedded tissue sections (4.0 μm thick) were deparaffinized in 3′ 10 minute washes of xylene, followed by 2′ 5 minute washes of 100.0% ethanol. The sections were treated with a pretreatment reagent (Abbott Molecular, Inc., Des Plaines, IL, USA) for 30–50 minutes at 80.0°C, after which the sections were treated with protease mixed with a protease buffer. The sections were hybridized using specific FISH probes. The results were analyzed using a fluorescence microscope (the AXIO Imager.D2) and AxioVision Microscopy Software for Windows, version 4.5 (Carl Zeiss AG, Oberkochen, Germany). In each case, 50 non-overlapping tumor cell nuclei were evaluated. Cases were considered to be MET amplification positive with a MET/CEN 7 ratio of ≥ 2.0 (truly amplified) and/or ≥ 5.0 copies of MET (high polysomy).

### Statistical analyses

In our study, categorical variables were analyzed using Pearson's chi-squared tests, except in instances where a small sample size of < 5 required the use of a Fisher's exact test. OS and PFS curves were calculated using the Kaplan-Meier method and compared by the log-rank test. Multivariate analysis of PFS was performed using the Cox proportional hazards model in which HRs and 95.0% CIs were derived for comparisons between the treatment subgroups of interest. All statistical analyses were conducted using Statistical Package for the Social Sciences for Windows software version 18.0 (SPSS Inc., Chicago, IL, USA). PFS curves were plotted using Stata for Windows software version 14.0 (StataCorp, College station, TX, USA). A two-sided *P* < 0.05 was considered statistically significant.

## SUPPLEMENTARY MATERIALS FIGURES AND TABLES



## Abbreviations

BMI: body mass index
CI: confidence interval
ECOG: Eastern Cooperative Oncology Group
EGFR-TKI: epidermal growth factor receptor-tyrosine kinase inhibitor
FISH: fluorescence *in situ* hybridization
HR: hazard ratio
IHC: immunohistochemical
ORR: objective response rate
OS: overall survival
PFS: progression-free survival
PS: performance status
