# Supplementary material for: Real-world experience of afatinib as a first-line therapy for advanced EGFR mutation-positive lung adenocarcinoma
Source: Oncotarget. 2017 Jul 26;8(52):90430–43. doi: 10.18632/oncotarget.19563 (PMC5685763; doi:10.18632/oncotarget.19563)
Supplement: Supplementary file 1 [file oncotarget-08-90430-s001.pdf]

Real-world experience of afatinib as a first-line therapy for advanced *EGFR* mutation-positive lung adenocarcinoma

SUPPLEMENTARY MATERIALS

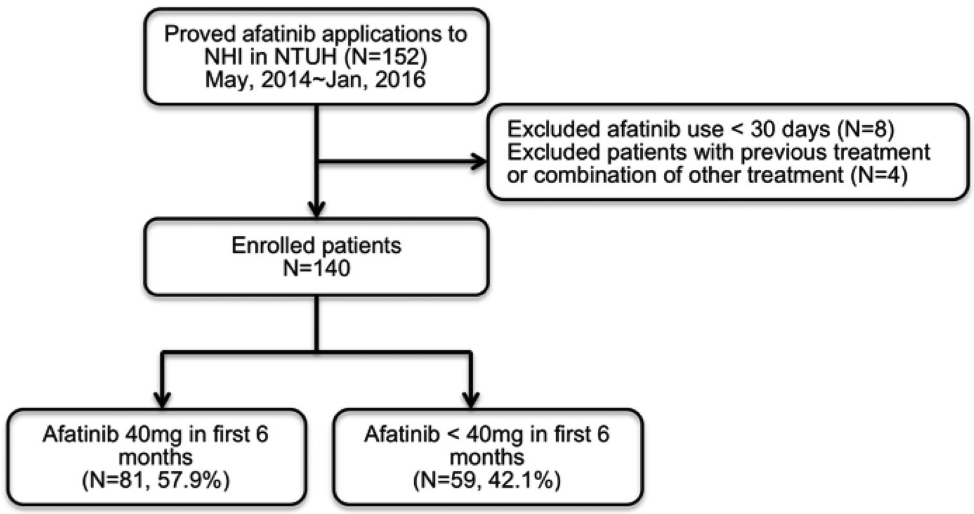

Supplementary Figure 1: Patient collection flow chart.

Supplementary Table 1: Clinical characteristics of patients in whom afatinib treatment was withdrawn (n = 7)

n	Age (y)/sex	SS	ECOG PS	BMI	cStage	EGFR mutation	Initial afatinib dose (mg)	TR	Time to DA (d)	Total afatinib (d)	Complication(s)	Substitute EGFR-TKI
1	82/F	NS	1	23.1	IV	p.L858R	40.0	PR	90	90	SR	Gefitinib
2	62/M	NS	0	25.2	IV	p.L858R	40.0	PR	30	30	IP	Erlotinib
3	58/F	NS	0	23.4	IV	p.L858R	40.0	PR	180	328	P	Erlotinib
4	52/F	NS	0	21.4	IV	p.L858R	40.0	SD	60	120	SR	Gefitinib
5	72/F	NS	1	26.9	IV	19DEL	30.0	PR	240	353	SR	Erlotinib
6	63/M	NS	1	23.5	IV	19DEL	40.0	PR	110	330	SR/OM	Erlotinib
7	74/F	NS	1	24.1	IV	20-INS	40.0	PR	30	45	OM	Gefitinib

BMI, body mass index; cStage, clinical stage; d, day; DA, dose adjustment; DEL, deletion; ECOG, Eastern Cooperative Oncology Group; EGFR, epidermal growth factor receptor; F, female; INS, insertion; IP, interstitial pneumonitis; M, male; NS, never smoked; OM, oral mucositis; P, paronychia; PR, partial response; PS, performance status; SD, stable disease; SR, skin rash; SS, smoking status; TKI, tyrosine kinase inhibitor; TR, treatment response; y, year

Supplementary Table 2: Dose adjustments of afatinib-treated patients in a real-world cohort

Variable	Afatinib-treated patients	
	Initial 40 mg (n = 98)	Initial 30 mg (n = 42)
Dose adjustment, n (%)		
Y	29 (29.6)	9 (21.4)
N	69 (70.4)	33 (78.6)
Dose adjustment sequence, n (%)		
Time to first dose reduction (days)		
< 180	18 (62.1)	7 (77.8)
≥ 180	11 (37.9)	2 (22.2)
Second dose adjustment		
Dose escalation	1 (3.4)	5 (55.6)
Dose reduction	4 (13.8)	1 (11.1)
No change	24 (82.8)	3 (33.3)

N, no; Y, yes

Supplementary Table 3: Treatment responses according to *EGFR* mutation status

***EGFR* mutation status**	**Patients, *n* (%)**	**Treatment response, *n* (%)**
Group 1 (classical mutation[s])	108 (77.1)	PR, 76 (70.4); SD, 26 (24.1); PD, 6 (5.5)
19DEL	81 (57.9)	PR, 55 (67.9); SD, 21 (25.9); PD, 5 (6.2)
p.L858R	24 (17.1)	PR, 18 (75.0); SD, 5 (20.8); PD, 1 (4.2)
p.L858R and 19DEL	3 (2.1)	PR, 3 (100.0)
Group 2 (complex mutation with classical mutation)	6 (4.3)	PR, 4 (66.7); SD, 2 (33.3)
p.L858R and p.T790M	4 (2.9)	PR, 2 (50.0); SD, 2 (50.0)
p.L858R and p.S768I	1 (0.7)	PR, 1 (100.0)
p.V765M and 19DEL	1 (0.7)	PR, 1 (100.0)
Group 3 (rare mutation with or without complex mutation)	26 (18.6)	PR, 14 (53.9); SD, 9 (34.6); PD, 3 (11.5)
p.L861Q	10 (7.2)	PR, 5 (50.0); SD, 3 (30.0); PD, 2 (20.0)
p.G719A	6 (4.3)	PR, 2 (33.3); SD, 4 (66.7)
20-INS	4 (2.9)	PR, 1 (25.0); SD, 2 (50.0); PD, 1 (25.0)
p.E709L and p.G719A	1 (0.7)	PR, 1 (100.0)
p.G719A and p.S768I	1 (0.7)	PR, 1 (100.0)
p.G719A and p.T790M	1 (0.7)	PR, 1 (100.0)
p.G719S and p.L747S	1 (0.7)	PR, 1 (100.0)
p.L861R and p.R776G	1 (0.7)	PR, 1 (100.0)
p.L861Q and p.S768I	1 (0.7)	PR, 1 (100.0)

DEL, deletion; INS, insertion; PD, progressive disease; PR, partial response; SD, stable disease.

Supplementary Table 4: The results of MET expression and amplifications in adequate tumor tissues (*n* = 17)

				MET in pre-afatinib tissues		MET in post-afatinib tissues			
n	**Age (y)/sex**	**SS**	***EGFR* mutation**	**IHC**	**FISH**	**IHC**	**FISH**	**TR**	**PFS of afatinib (m)**
1	66/M	CS	p.L858R and p.T790M	3+	Negative	3+	Positive	PR	5.0
2	70/F	NS	p.E709K and p.G719A	2-3+	Negative	0-1+		PR	10.5
3	44/M	CS	19DEL	2+	Negative	3+	Negative	SD	11.8
4	64/M	NS	p.L858R	2+	Negative	0-1+		PR	19.0
5	57/F	NS	19DEL	2+	Negative	0		PR	11.0
6	51/M	NS	19DEL	0-1+		3+	Negative	PR	9.0
7	58/M	CS	p.L858R	0		2+	Negative	SD	9.3
8	58/M	NS	p.L858R and p.T790M	0-1+		3+	Negative	SD	4.0
9	67/M	CS	p.G719A	1+		3+	Negative	SD	6.0
10	61/F	NS	p.L858R	1+		1+		PR	8.3
11	49/F	NS	p.L858R	1+		1+		PR	7.0
12	68/F	NS	20-INS	0-1+		0-1+		SD	3.7
13	67/M	CS	19DEL	0-1+		1+		SD	8.1
14	45/F	NS	19DEL	0		0		PR	8.7
15	63/F	NS	19DEL	1+		0-1+		PR	17.3
16	54/M	CS	p.L858R	0-1+		0-1+		PR	12.8
17	54/F	NS	19DEL	NA		2+	Negative	PD	3.0

CS, current smoker; DEL, deletion; EGFR, epidermal growth factor receptor; F, female; FISH, fluorescence *in situ* hybridization; IHC, Immunohistochemical; INS, insertion; M, male; m, months; NA: not available; NS, never smoked; PD, progressive disease; PR, partial response; PFS, progression-free survival; SD, stable disease; SS, smoking status; TKI, tyrosine kinase inhibitor; TR, treatment response
